# Commentary: Accelerating spiking neural network simulations with PymoNNto and PymoNNtorch

**DOI:** 10.3389/fninf.2024.1446620

**Published:** 2024-10-23

**Authors:** Hans Ekkehard Plesser

**Affiliations:** ^1^Department of Data Science, Faculty of Science and Technology, Norwegian University of Life Sciences, Ås, Norway; ^2^Institute for Advanced Simulation (IAS-6), Research Centre Jülich, Jülich, Germany; ^3^Käte Hamburger Kolleg: Cultures of Research (c:o/re), RWTH Aachen University, Aachen, Germany

**Keywords:** spiking neural network (SNN), comparison, simulator, efficient implementation, exact integration

## 1 Introduction

In a recent paper introducing the PymoNNto and PymoNNtorch simulators, Vieth et al. (2024) compare their own simulators to several publicly available simulation codes using two different neuronal network models. They report that “the native NEST implementation consistently generates slightly fewer spikes in comparison with the other simulators” for their LIF neuron network model (see their Supplementary Figure S2) but do not investigate this issue beyond confirming that the discrepancy is not caused by the use of double-precision numerics in NEST.

This commentary shows that the observed difference in firing rate is due to an unsuitable numerical integration scheme used in the other simulators while NEST creates the correct model dynamics.

## 2 Results

Vieth et al. (2024) study a weakly coupled all-to-all network of *K* excitatory neurons driven by individual random input. The plasticity mechanism in the network is not relevant to the numerical issues investigated here and thus left out. Generalizing the equations of the original paper slightly, the membrane potential of an individual neuron *k* is given by


(1) $$\begin{array}{l} {\dot{v}}^{\left(k\right)}\left(t\right)=-\frac{v^{\left(k\right)}\left(t\right)}{\tau }+\frac{I_{\text{ext}}^{\left(k\right)}\left(t\right)+I_{\text{syn}}^{\left(k\right)}\left(t\right)}{C} \end{array}$$

with membrane time constant τ and capacitance *C*. The membrane potential is reset to *v*^(*k*)^ = 0 when *v*^(*k*)^ = θ and there is no refractory time. The external input current is defined by


(2) $${\dot{I}}_{\text{ext}}^{\left(k\right)}(t)=\left(U[I_{\text{min}},I_{\text{max}})-I\right)\delta (t-jh)$$

is a piecewise constant current with a new amplitude chosen from a uniform distribution at fixed time intervals *h*. The recurrent synaptic input is


(3) $$\begin{array}{l} I_{\text{syn}}^{\left(k\right)}\left(t\right)=\underset{l,i}{\sum }w_{kl}\delta \left(t-t^{\left(l,i\right)}-d\right) \end{array}$$

with uniformly distributed synaptic weights $w_{kl}\sim U\left[0,1/K\right)\text{mV}$, the synaptic delay *d*, and *t*^(*l, i*)^ representing the time of the *i*^th^ spike fired by neuron *l*.

For the parameters used by Vieth et al. (2024),^1^ the mean input current is $\mu =\frac{1}{2}\text{pA}$ with variance $\sigma^{2}=\frac{1}{12}{\text{pA}}^{2}$, and the average synaptic weight $w=\frac{1}{2K}\text{mV}=5\cdot 10^{-5}\text{mV}$. In this regime, spiking activity is mainly driven by external input with an average firing rate between 100ms and 295ms of 11.6sp/s for the full model and 9.9sp/s without any connections.

We consider first an individual neuron driven by external noise only. Dropping unnecessary subscripts and discretizing the dynamics on the fixed time grid *t*_*j*_ = *jh* at which the input current takes on new random values *I*_*j*_, Equation 1 becomes


(4) $$\begin{array}{l} v_{j+1}=\beta v_{j}+\alpha I_{j}. \end{array}$$

The coefficients α and β depend on the numerical integration method chosen.

Equation 4 will provide the *exact* solution of Equations 1 and 2 if we use the exact integration method (Rotter and Diesmann, 1999) for which


(5) $$\begin{array}{l} \alpha =\frac{\tau }{C}\left(1-e^{-h/\tau }\right)\approx 0.952 \end{array}$$

(6) $$\begin{array}{l} \beta =e^{-h/\tau }\approx 0.905. \end{array}$$

In the specific case of piecewise constant input over the integration step as in the given model, this method is equivalent to the exponential integration by MacGregor (1987, Ch. 14.C.5). We will use this solution as reference.

The coefficients for the forward Euler method are


(7) $$\begin{array}{l} \alpha =\frac{h}{C}=1.0 \end{array}$$

(8) $$\begin{array}{l} \beta =1-\frac{h}{\tau }=0.9. \end{array}$$

For first-passage time problems for the process defined by Equation 4 in combination with a spiking threshold, closed-form solutions exist for noise symmetric to the origin (Larralde, 2004), while only bounds are known for more general cases as studied here (Novikov and Kordzakhia, 2008).

We thus consider the free membrane potential in the absence of a threshold. Assuming *v*_0_ = 0, we obtain by repeated application of the update equation


(9) $$\begin{array}{l} v_{j+1}=\alpha \overset{j}{\underset{k=0}{\sum }}\beta^{k}I_{k}, \end{array}$$

where we have renumbered the random currents *I*_*k*_, exploiting their independence. Averaging over currents and taking the limit *j* → ∞, we obtain the mean membrane potential and its standard deviation


(10) $$\begin{array}{l} \langle v\rangle =\frac{\alpha }{1-\beta }\mu =\frac{\tau }{C}\mu =5\text{ mV} \end{array}$$

(11) $$\begin{array}{l} \sqrt{\langle \Delta v^{2}\rangle }=\frac{\alpha }{\sqrt{1-\beta^{2}}}\sigma^{2}\\ \;=\{\begin{array}{ll} \frac{\tau \sigma }{C}\sqrt{\frac{1-e^{-h/\tau }}{1+e^{-h/\tau }}}\approx 0.645\text{ mV} & \text{(exact integration)}\\ \frac{\tau \sigma }{C}\sqrt{\frac{h}{2\tau -h}}\approx 0.662\text{ mV} & \text{(forward Euler) }. \end{array} \end{array}$$

The broader distribution of the free membrane potential in the case of forward Euler integration (8.1% more probability mass above threshold θ) suggests that neurons will fire more frequently. This is supported by simulations below.

We confirm this observation quantitatively through numerical Markov analysis. This analysis is in principle continuous in time, but as the noise in the network model switches in intervals *h*, we consider only time points at which the noise changes. Given *v*_*k*_, the smallest possible *v*_*k*+1_ is obtained for *I* = *I*_min_ during the time step and the largest for *I* = *I*_max_. In between the maximum and minimum possible values, any value of *v*_*k*+1_ is attained with equal probability. We can thus write the free transition probability for the membrane potential as


(12) $$\hat{p}(v^{\prime }|v)\;=\{\begin{array}{ll} 0 & \text{if }v^{\prime }<\alpha I_{\text{min}}+\beta v\Leftrightarrow v>(v^{\prime }-\alpha I_{\text{min}})/\beta \\ \frac{1}{\tau (I_{\text{max}}-I_{\text{min}})} & \text{else}\\ 0 & \text{if }v^{\prime }>\alpha I_{\text{max}}+\beta v\Leftrightarrow v<(v^{\prime }-\alpha I_{\text{max}})/\beta \end{array}$$

(13) $$\begin{array}{l} =\frac{1}{\tau (I_{\text{max}}-I_{\text{min}})}[\Theta \left(v-\frac{v^{\prime }-\alpha I_{\text{min}}}{\beta }\right)\\ \;-\Theta \left(v-\frac{v^{\prime }-\alpha I_{\text{max}}}{\beta }\right)] \end{array}$$

where Θ(*x*) is the Heaviside step function. To include the effect of the spiking threshold θ, we define


(14) $$p(v^{\prime }|v)=\{\begin{array}{ll} \hat{p}(0|v)+\int_{\theta }^{\infty }\hat{p}(v^{\prime }|v)dv^{\prime } & v^{\prime }=0\\ 0 & v^{\prime }>\theta \\ \hat{p}(v^{\prime }|v) & \text{else.} \end{array}$$

Here, the integral in the first clause describes neurons re-inserted at the reset potential after spiking, and the corresponding probability is removed for superthreshold *v*′ by the second clause.

If *q*_*k*_(*v*) is the membrane potential distribution at time step *k*, then the distribution at step *k*+1 is given by


(15) $$q_{k+1}(v^{\prime })=\int_{-\infty }^{\infty }p(v^{\prime }|v)q_{k}(v)dv\;.$$

It will converge to the stationary membrane potential distribution *q*^(∞)^(*v*) under reasonable assumptions about *p*(*v*′|*v*) (Lasota and Mackey, 1994). The firing probability for an interval *h* is given by the integral term in Equation 14, yielding the steady-state firing rate


(16) $$r^{\left(\infty \right)}=h\int_{-\infty }^{\theta }\left[p(0|v)-\hat{p}(0|v)\right]q^{\left(\infty \right)}(v)dv\;.$$

As no closed-form solution is available for these equations, we discretize the transition probability as a Markov matrix *M*. This matrix has a single eigenvector with eigenvalue 1 which corresponds to *q*^(∞)^(*v*) up to normalization (Feller, 1970; von Mises, 1964).

**Figure 1:**
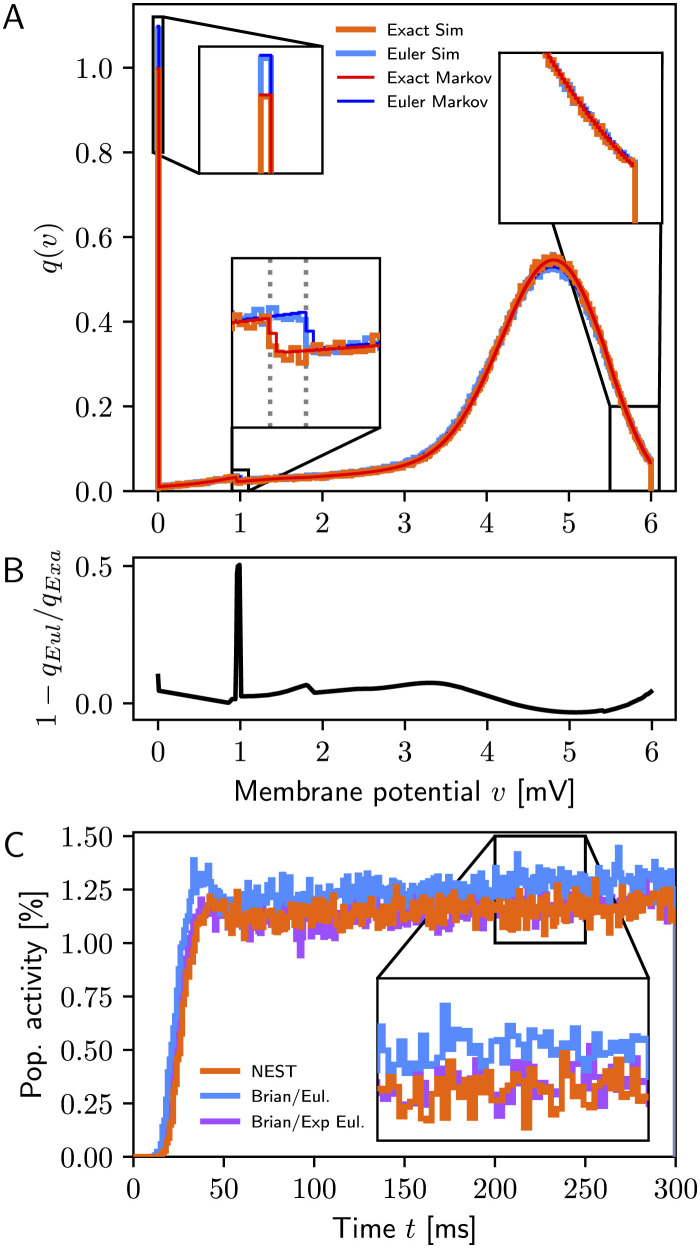
**(A)** Stationary membrane potential distributions *q*^(∞)^(*v*) for exact integration (simulation: orange, Markov analysis: red) and forward Euler (simulation: light blue, Markov analysis: dark blue). Simulation data are for a single simulation of 1, 000 s duration; bin width 0.01 mV. The dotted lines in the bottom-left inset mark α = 0.952 for exact integration and α = 1.0 for forward Euler, respectively, indicating the difference in the first update step after reset to *v* = 0mV. **(B)** Relative difference in membrane potential distributions between exact integration and forward Euler. The peak near 1 mV is due to the difference in the first step after reset. **(C)** Population activity (1ms bins) for simulations with NEST (orange) and Brian2 using delta synapses (blue: forward Euler, purple: exponential Euler); data from a single simulation with 10,000 neurons.

In the absence of recurrent connections, Figure 1A shows excellent agreement between simulations and Markov analysis (top) for exact integration and forward Euler, respectively, whereas Figure 1B displays clear differences in the membrane potential distributions between the two models. We obtain from the Markov analysis for exact integration *r*^(∞)^ = 9.93sp/s vs. 9.96 ± 0.04sp/s from simulations and for forward Euler *r*^(∞)^ = 10.92sp/s vs. 10.93 ± 0.04sp/s. This difference of approximately 1sp/s between exact integration and forward Euler agrees closely with the disparity reported by Vieth et al. (2024, Supplementary Figure S2).

When recurrent connections are included and simulations performed using the original authors' code (PesarAmmehZA and Vieth, 2024, git hash c9c59f0), simulation results differ from results obtained with NEST even if using the exponential Euler method in Brian2. Analysis of their code (Brian_LIF.py) revealed that this neuron model used exponential post-synaptic currents instead of delta pulses. Once this was corrected, results obtained with Brian2 with exponential integration were consistent with NEST results as shown in Figure 1C.

Careful analysis of STDP in a network of 1,000 neurons revealed errors in both NEST and Brian2. NEST missed approximately 10% of weight increases, which could be traced to an error in NESTML code generation which has been fixed.^2^ For Brian2, a small number of synapses experienced up to seven STDP events, while at most three are expected. Details are given in the Supplementary material.

## 3 Conclusion

Based on the exact solution (Rotter and Diesmann, 1999) to the model defined by Equations 1 and 2, the analysis above confirms that the discrepancy in firing rates between NEST and the other simulation codes observed by Vieth et al. (2024) is due to the use of the unsuitable forward Euler method in the latter codes: using this method to integrate the membrane potential evolution actually changes the model under study. Indeed, inserting α and β for exact integration into Equations 7 and 8 and solving for τ and *C* shows that the forward Euler method solved the model for τ≈10.5 ms and *C*≈1.05 pF. Only NEST, the “odd one out,” actually simulated the model as defined mathematically.

Interestingly, the investigation of the discrepancy observed by Vieth et al. (2024) helped us to uncover a subtle bug in NESTML. This shows that careful comparison of results by different simulators provides mutual benefits.

The observed difference in firing rates of approximately 10% most likely will not have more than a 10% effect on the simulation runtimes measured by Vieth et al. (2024) and thus will not affect their overall conclusions. Nonetheless, I find it important to point out that benchmarks are meaningless, unless one defines a desired level of precision (Morrison et al., 2007) and confirms that the simulation codes compared produce statistically equivalent results (see, e.g., Van Albada et al., 2018).

Finally, I would like to question the choice of benchmark model as such. The LIF neuron network model defined by Vieth et al. (2024) is peculiar in that it is a purely excitatory all-to-all network with a plasticity rule that only allows synapses to become stronger. The model can therefore only be benchmarked over a limited simulation time before run-away excitation leads to excessive spike rates. Balanced networks such as the two-population model by Brunel (2000) can, in contrast, be simulated for arbitrary periods of time and usually in different dynamic regimes. Furthermore, comparability with other studies might benefit from using the cortical microcircuit model by Potjans and Diesmann (2014) as a benchmark case, as this model has become widely used as a reference model in recent years (Knight and Nowotny, 2018; Van Albada et al., 2018; Golosio et al., 2021; Kauth et al., 2023).

## Footnotes

^1^*K* = 10^4^, τ = 10 ms, *C* = 1 pF, *h* = *d* = 1 ms, *I*_min_ = 0 pA, *I*_max_ = 1 pA, θ = 6 mV.

^2^ https://github.com/nest/nestml/issues/1057
