# Supplementary material for: Commentary: Accelerating spiking neural network simulations with PymoNNto and PymoNNtorch
Source: Front Neuroinform. 2024 Oct 23;18:1446620. doi: 10.3389/fninf.2024.1446620 (PMC11537845; doi:10.3389/fninf.2024.1446620)
Supplement: Supplementary file 1 [file Data_Sheet_1.pdf]

Supplementary Material for H. E. Plesser (2024): Commentary on Vieth et al (2024)

This notebook provides code, simulations and analysis for the results reported in my *Comment* on the work by Vieth et al (2024). It is available at https://github.com/heplesser/comment_vieth_code together with scripts used.

In [1]:
```
import random
import math
import numpy as np
import matplotlib.pyplot as plt
import subprocess
import pandas as pd
import pickle
from scipy.stats import norm, binom
from pathlib import Path
```

The model

As pointed out in the *Comment*, we can limit ourselves to the dynamics of an isolated neuron to understand the discrepancy in simulation results between NEST and other simulators for the *Simple* network studied by Vieth and colleagues.

Ignoring recurrent input and focusing on a single neuron, the membrane potential in this model evolves according to

$$\frac{dv}{dt} = -\frac{v}{\tau} + \frac{I}{C} \tag{1}$$

$$\dot{I} = (\mathcal{U}[I_{\min}, I_{\max}) - I)\, \delta(t - jh) \tag{2}$$

When the membrane potential reaches the threshold $V = \theta$, a spike is emitted and the membrane potential reset to 0mV. There is no refractory time. The input current changes in intervals of $h = 1$ms, and this is the time step used for numerical integration.

Parameters used by Vieth et al are

In [2]:
```
tau   = 10  # ms
C     =  1  # pF
I_min =  0  # pA
I_max =  1  # pA
theta =  6  # mV
h     =  1  # ms
```

Time-discrete solution

For any ODE solution scheme linear in the membrane potential and input, we can write the update equation as

$$v_{k+1} = \beta v_k + \alpha I_k \ .$$

This applies also to higher-order schemes such as RK4.

In particular, we have for exact (exponential) integration

$$\alpha = \frac{\tau}{C}\left(1 - e^{-h/\tau}\right), \qquad \beta = e^{-h/\tau}$$

and for forward Euler integration

$$\alpha = \frac{h}{C}, \qquad \beta = 1 - \frac{h}{\tau} .$$

Numerical values of integration coefficients

In [3]:
```
alpha_exact, beta_exact = - tau / C * math.expm1(-h/tau), math.exp(-h/tau)
alpha_euler, beta_euler = h / C, 1 - h / tau
```

In [4]:
```
print(f"Exact: alpha = {alpha_exact:6.4f}, beta = {beta_exact:6.4f}")
print(f"Euler: alpha = {alpha_euler:6.4f}, beta = {beta_euler:6.4f}")
```

```
Exact: alpha = 0.9516, beta = 0.9048
Euler: alpha = 1.0000, beta = 0.9000
```

Actual model parameters for the Euler case

$$\tau_{\text{Euler}} = \frac{h}{1 - \beta_{\text{Exact}}} \tag{3}$$

$$C_{\text{Euler}} = \frac{h}{\alpha_{\text{Exact}}} \tag{4}$$

In [5]:
```
tau_euler = h / (1 - beta_exact)
C_euler = h / alpha_exact
```

In [6]:
```
print(f"tau_euler = {tau_euler:5.2f} ms")
print(f"C_euler   = {C_euler:5.2f} pF")
```

```
tau_euler = 10.51 ms
C_euler   =  1.05 pF
```

Mean and standard deviation of the free membrane potential

Mean and standard deviation of the noise input

In [7]:
```
mu_I = (I_min + I_max) / 2
sigma_I = math.sqrt((I_max - I_min)**2/12)

print(f"mu = {mu_I:6.4f}pA, sigma = {sigma_I:6.4f}pA")
```

```
mu = 0.5000pA, sigma = 0.2887pA
```

Free membrane potential under exact integration

Compute mean and standard deviation and survival probability $S(v)$, i.e., the probability mass of the free membrane potential distribution above threshold θ.

In [8]:
```
V_mean_exact = alpha_exact / (1 - beta_exact) * mu_I
V_std_exact = alpha_exact / math.sqrt(1 - beta_exact**2) * sigma_I
S_exact = norm.sf(theta, loc=V_mean_exact, scale=V_std_exact)
V_mean_euler = alpha_euler / (1 - beta_euler) * mu_I
V_std_euler = alpha_euler / math.sqrt(1 - beta_euler**2) * sigma_I
S_euler = norm.sf(theta, loc=V_mean_exact, scale=V_std_euler)
```

In [9]:
```
print(f"Exact <V>           = {V_mean_exact:6.4f}mV")
print(f"Exact <(DV)^2>^1/2 = {V_std_exact:6.4f}mV")
print(f"Exact S(theta)      = {S_exact:6.4f}")
print()
```

```
print(f"Euler <V>           = {V_mean_euler:6.4f}mV")
print(f"Euler <(DV)^2>^1/2 = {V_std_euler:6.4f}mV")
print(f"Euler S(theta)     = {S_euler:6.4f}")
```

```
Exact <V>           = 5.0000mV
Exact <(DV)^2>^1/2 = 0.6452mV
Exact S(theta)     = 0.0606

Euler <V>           = 5.0000mV
Euler <(DV)^2>^1/2 = 0.6623mV
Euler S(theta)     = 0.0655
```

In [10]:

```
print(f"Excess survival prob for Euler: {(S_euler/S_exact-1)*100:.2f}%")
```

```
Excess survival prob for Euler: 8.15%
```

Numerical simulation

Simultaneous integration using both methods

In [11]:

```
def sim(t_max):
    """
    Returns t, V_exact, V_euler, spikes_exact, spikes_euler
    """

    t = np.arange(0, t_max + 0.5 * h, h)
    V_exact = np.zeros_like(t)
    V_euler = np.zeros_like(t)
    s_exact = []
    s_euler = []

    for ix in range(1, len(t)):
        # same input current for both methods
        I = I_min + (I_max - I_min) * random.random()

        V_exact[ix] = beta_exact * V_exact[ix-1] + alpha_exact * I
        if V_exact[ix] >= 6.0:
            V_exact[ix] = 0
            s_exact.append(t[ix])

        V_euler[ix] = beta_euler * V_euler[ix-1] + alpha_euler * I
        if V_euler[ix] >= theta:
            V_euler[ix] = 0
            s_euler.append(t[ix])

    return t, V_exact, V_euler, np.array(s_exact), np.array(s_euler)
```

Perform simulations in Python

The code below simulates 10000 individual neurons five times over and reports firing rates reported. Firing rates are computed for the [100, 300] ms interval to obtain the firing rate for the stationary state.

In [12]:

```
random.seed(12345)

t_sim = 300
t_min = 100
n_sims = 10000

rates_exact, rates_euler = [], []
for _ in range(5):
    num_exact, num_euler = [], []
    for _ in range(n_sims):
        t, V_exact, V_euler, s_exact, s_euler = sim(t_sim)
        num_exact.append(len(s_exact[s_exact > t_min]))
        num_euler.append(len(s_euler[s_euler > t_min]))

    rates_exact.append(sum(num_exact) / n_sims / ( t_sim - t_min ) * 1000)
    rates_euler.append(sum(num_euler) / n_sims / ( t_sim - t_min ) * 1000)
```

Resulting firing rates

In [13]:
```
print(f'Rate Exact: {np.mean(rates_exact):6.2f} ± {np.std(rates_exact):4.2f} sp/s ')
print(f'Rate Euler: {np.mean(rates_euler):6.2f} ± {np.std(rates_euler):4.2f} sp/s ')
```

```
Rate Exact:   9.96 ± 0.04 sp/s
Rate Euler:  10.93 ± 0.04 sp/s
```

Markov chain model

Given v_k, the smallest possible v_{k+1} is obtained for $I_k = I_{\text{min}}$ during the time step and the largest for $I_k = I_{\text{max}}$. In between the maximum and minimum possible values, any value of v_{k+1} is attained with equal probability.

We can thus write the transition probability for the membrane potential as

$$\hat{p}(v'|v) = \begin{cases} 0 & \text{if} \quad v' < \alpha I_{\text{min}} + \beta v \Leftrightarrow v > (v' - \alpha I_{\text{min}})/\beta \\ \frac{1}{\tau(I_{\text{max}} - I_{\text{min}})} & \text{else} \\ 0 & \text{if} \quad v' > \alpha I_{\text{max}} + \beta v \Leftrightarrow v < (v' - \alpha I_{\text{max}})/\beta \end{cases} \tag{5}$$

$$= \frac{1}{\tau(I_{\text{max}} - I_{\text{min}})} \left[\Theta\left(v - \frac{v' - \alpha I_{\text{min}}}{\beta}\right) - \Theta\left(v - \frac{v' - \alpha I_{\text{max}}}{\beta}\right) \right] . \tag{6}$$

To include the effect of the spiking threshold θ, we define

$$p(v'|v) = \begin{cases} \hat{p}(0|v) + \int_\theta^\infty \hat{p}(v'|v) dv' & v' = 0 \\ 0 & v' > \theta \\ \hat{p}(v'|v) & \text{else.} \end{cases}$$

If $q_k(v)$ is the membrane potential distribution at time step k, then the distribution at step $k+1$ is given by

$$q_{k+1}(v') = \int_{-\infty}^{\infty} p(v'|v) q_k(v) dv .$$

Discrete Markov chain

We discretize the transition operator $p(v'|v)$ and the membrane potential distribution $q_k(v)$ evenly along the voltage axis between 0 and θ to solve for the stationary distribution numerically.

In [14]:
```
def markov(kind, dv=0.01):
    """
    Input "kind" is either "exact" or "euler".

    Returns the stationary distribution, the transition matrix and the voltage axis.

    dv      : discretization of matrix, in mV
    delta_v : membrane potential lift due to recurrent input in single time step
    """

    if kind == 'exact':
        alpha = alpha_exact
        beta = beta_exact
    elif kind == 'euler':
        alpha = alpha_euler
        beta = beta_euler
    else:
        raise ValueError(f'Unknow kind "{kind}"')

    I_min, I_max = 0, 1
```

```
    # Create a voltage axis that will contain all non-vanishing parts
    # of the free membrane potential distribution.
    vv = np.arange(0, max(V_mean_exact, theta) + 6 * V_std_exact, dv)

    # Find location of threshold
    ix_theta = sum(vv < theta)

    # Create Markov matrix for free membrane potential distribution (normalized below)
    # This matrix extends beyond the threshold in order to compute the superthreshold
    # elements that then are integrated to represent re-injection after firing.
    #
    # Each element of M represents the probability that if the membrane potential is in
    # [v_from, v_from+dv), then it will be in [v_to, v_to+dv) in the next step. The smallest
    # possible voltage step is from v_from+dv -> v_to, while the largest is v_from -> v_to + dv
    # and we calculate the corresponding input currents below.
    #
    # M is first filled with relative probabilities and normalized columnwise below.
    M = np.zeros((len(vv), len(vv)))
    v_from_min, v_to_min = np.meshgrid(vv, vv)
    v_from_max = v_from_min + dv
    v_to_max = v_to_min + dv
    I_from_max_to_min = np.clip((v_to_min - beta * v_from_max) / alpha, I_min, I_max)
    I_from_min_to_max = np.clip((v_to_max - beta * v_from_min) / alpha, I_min, I_max)
    M = abs(I_from_min_to_max - I_from_max_to_min)

    # Sum superthreshold values representing spiking and add
    # to row representing transition to 0 ≤ v < dv
    p_spike = M[ix_theta:, :].sum(axis=0)
    M[0, :] += p_spike

    # Restrict matrix and voltage axis to subthreshold parts
    M = M[:ix_theta, :ix_theta]
    vv = vv[:ix_theta]

    # Normalize matrix columnwise
    M /= M.sum(axis=0)

    # Compute eigenvalues, largest will come first and should be 1
    lam, ev = np.linalg.eig(M)
    assert np.isclose(lam[0], 1), "Largest eigenvector == 1 expected"

    # Stationary distribution is normalized first eigenvector
    phi = np.abs(ev[:, 0]) / np.sum(np.abs(ev[:, 0]))

    return phi, M, vv
```

Evaluate for both rules

In [15]:

```
phi_exact, M_exact, vv_exact = markov('exact')
phi_euler, M_euler, vv_euler = markov('euler')
```

Illustrate evolution of membrane potential

Starting from a $q_0(V) = \delta(V)$, the membrane potential evolves quickly towards a Gaussian-like shape while still too far from the threshold to feel its effects and then converges rather quickly to the stationary distribution.

In [16]:

```
plt.figure(figsize=(10, 3))

q = np.zeros_like(vv_exact)
q[0] = 1
for i in range(51):
    q = M_exact @ q
    if i < 10 or (i+1) % 10 == 0:
        plt.plot(vv_exact, q, label=f'q_{i+1}');

plt.plot(vv_exact, phi_exact, 'k-', lw=5, alpha=0.2, label='q^(oo)');
```

```
plt.legend(fontsize='xx-small');
plt.xlabel('V [mV]');
plt.ylabel('p(V)');
plt.title('Evolution of membrane potential distribution (exact integration)');
```

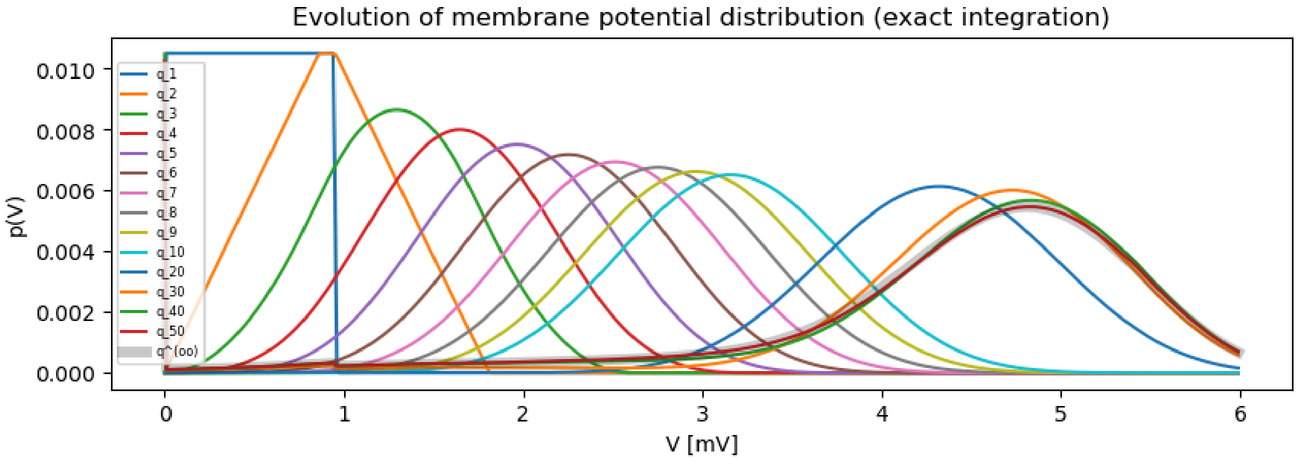

Compute firing rates from Markov analysis in absence of recurrent input

The stationary-state firing rate is determined by the stationary membrane potential distribution and the integral term in the first clause of Eq. 15 in the paper. This integral term will have non-zero contributions only for v sufficiently close to the threshold that v can reach θ in a single step of duration h. To extract this part of $p(v' = 0|v)$ from the matrix `M`, we zero out the first part of the first row of the matrix, i.e., the contribtions of $\hat{q}(0|v)$. Multiply by 1000 to convert from per ms to per s.

In [17]:
```
def markov_rate(M, phi_inf, dv):
    M0 = M[0, :].copy()
    lim_blank = int(1 / dv)    # blank first mV
    M0[:lim_blank] = 0
    return M0 @ phi_inf * 1000
```

In [18]:
```
rate_exact_markov = markov_rate(M_exact, phi_exact, vv_exact[1])
rate_euler_markov = markov_rate(M_euler, phi_euler, vv_euler[1])

print(f'Rate Exact: {rate_exact_markov:6.2f} sp/s')
print(f'Rate Euler: {rate_euler_markov:6.2f} sp/s')
```

```
Rate Exact:   9.93 sp/s
Rate Euler:  10.92 sp/s
```

Simulations with NEST and Brian2

The following simulations have been performed with

- NEST 3.7
- NESTML 7.0.2
- Brian2 2.6.0
- Code by Vieth et al (2024) from https://github.com/saeedark/Fast-SNN-PymoNNto-rch/tree/main at c9c59f0c6cc2cb3f52875ea1615e4fc9acdd7904.

Vieth et al (2024) used a developer version of NESTML downstream of NESTML 5.3.0. The precise version used (Git hash) is not available from their paper. I use NESTML 7.0.2 here, which requires minor changes to the input specification in the NESTML code from

```
input:
    spikes mV <- spike
    I_e pA <- continuous
```

to

```
input:
    spikes <- spike
    I_e pA <- continuous
```

I also extended Python scripts `Benchmarks/Simples/{brian_LIF,nest_native_LIF,globparams}.py` to support disabling connectivity entirely, to disable STDP, to change the Brian integration rule and to record spikes and optionally weights before and after simulation.

In [19]:
```python
def run_sims(code, n_runs, n_neurons, *args):
    """
    Simulate network by running given code.

    code      - name of Python script to run; must write output to tmpdata
    n_runs    - number of simulations to run
    n_neurons - network size
    args      - these options can be passed
                no_plot - do not plot at end of simulation
                no_conn - do not create any connections
                no_stdp - disable STDP by setting stdp_speed to 0
                rec_weights - record all weights before and after simulation
                psc_delta   - use delta synapses instead of exponential in Brian
                exponential_euler - if given, use this method instead of euler in Brian

    Returns list of lists of spike times and neuron ids and optionally, initial and final weights.
    """

    if code.startswith('nest_'):
        fb = (f"nest_native_LIF_{n_neurons}_Conn_{'no_conn' not in args}",
              f"_STDP_{'no_stdp' not in args}")
    else:
        EE = 'exponential_euler'
        fb = (f"brian_LIF_Delta_{'psc_delta' in args}_{EE if EE in args else 'euler'}",
              f"_{n_neurons}_Conn_{'no_conn' not in args}_STDP_{'no_stdp' not in args}")
    base = Path('Benchmarks/Simple/')
    cmd = ['python', base / code, str(n_neurons), 'no_plot']
    cmd.extend(args)
    spikes_t = []
    spikes_gid = []
    w_ini = []
    w_post = []
    w_src = []
    w_tgt = []
    wrec = []
    for _ in range(n_runs):
        subprocess.check_call(cmd)
        s = pd.read_csv(next(Path('tmpdata').glob(f'{fb}_spikes.dat')), comment='#', sep='\t')
        spikes_t.append(s.times)
        spikes_gid.append(s.senders)
        if 'rec_weights' in args:
            w = pd.read_csv(next(Path('tmpdata').glob(f'{fb}_weights.dat')), comment='#', sep='\t')
            w_ini.append(w.w_ini)
            w_post.append(w.w_post)
            if code.startswith('nest_'):
                w_src.append(w.source)
                w_tgt.append(w.target)
                wr = pd.read_csv(next(Path('tmpdata').glob(f'{fb}_wr.dat')), comment='#', sep='\t')
                wrec.append(wr)

    return {'spikes_t': spikes_t, 'spikes_id': spikes_gid, 'w_ini': w_ini, 'w_post': w_post,
            'w_src': w_src, 'w_tgt': w_tgt, 'wrec': wrec}
```

In [20]:

```python
def run_simset(n_runs, n_size, *args):
    """
    Run set of simulations for NEST, Brian with Euler and Exponential Euler,
    with exponential and delta synapses.

    Same parameters as for run_sims(), except that args shall not contain
    exponential_euler or psc_delta.

    Returns dictionary of results and writes results to data in pickled form.
    """

    nest = run_sims('nest_native_LIF.py', n_runs, n_size, *args)
    brian_exp_euler = run_sims('brian_LIF.py', n_runs, n_size, *args)
    brian_exp_expeuler = run_sims('brian_LIF.py', n_runs, n_size, 'exponential_euler', *args)
    brian_delta_euler = run_sims('brian_LIF.py', n_runs, n_size, 'psc_delta', *args)
    brian_delta_expeuler = run_sims('brian_LIF.py', n_runs, n_size, 'psc_delta',
                                    'exponential_euler', *args)

    res = {'nest': nest,
           'brian_exp_euler': brian_exp_euler, 'brian_exp_expeuler': brian_exp_expeuler,
           'brian_delta_euler': brian_delta_euler, 'brian_delta_expeuler': brian_delta_expeuler}

    fn = f"sim_{n_size}_Conn_{'no_conn' not in args}_STDP_{'no_stdp' not in args}.pkl"
    pickle.dump(res, open(Path('data') / fn, 'wb'))
```

NEST & Brian simulation

Next cell takes long to run, data are loaded form pickle in following cell

In [21]:

```python
if False:
    run_simset(5, 10000)                # full size, complete network
if False:
    run_simset(5, 10000, 'no_stdp')     # full size, complete network, no plasticity
if False:
    run_simset(5, 10000, 'no_conn')     # full size, no connections
```

Load simulation results from above

In [22]:

```python
full = pickle.load(open('data/sim_10000_Conn_True_STDP_True.pkl', 'rb'))
no_stdp = pickle.load(open('data/sim_10000_Conn_True_STDP_False.pkl', 'rb'))
no_conn = pickle.load(open('data/sim_10000_Conn_False_STDP_True.pkl', 'rb'))
```

Firing rates from simulations

Compute rates over $[t_{\min}, t_{\max}]$ with $t_{\max}, t_{\text{sim}}$ to avoid initial transient and differences between simulators on what gets recorded from last time step.

In [23]:

```python
def compute_rates(data, t_min=100, t_max=295, n_size=10000):
    mn, std = {}, {}
    for name, res in data.items():
        rates = [len(d[(t_min < d) & (d <= t_max)]) / ((t_max-t_min)/1000) / n_size
                 for d in res['spikes_t']]
        mn[name] = np.mean(rates)
        std[name] = np.std(rates)

    return {'mean': mn, 'std': std}

def print_rates(res):
    for name in res['mean'].keys():
        print(f"Firing rate {name:20}: {res['mean'][name]:5.2f} ± {res['std'][name]:4.2f} sp/s")
```

Full model

In [24]:
```
rates_full = compute_rates(full)
print_rates(rates_full)
```

```
Firing rate nest                 : 11.56 ± 0.07 sp/s
Firing rate brian_exp_euler      : 12.86 ± 0.05 sp/s
Firing rate brian_exp_expeuler   : 13.79 ± 0.06 sp/s
Firing rate brian_delta_euler    : 12.62 ± 0.04 sp/s
Firing rate brian_delta_expeuler: 11.66 ± 0.07 sp/s
```

Model without STDP

In [25]:
```
rates_no_stdp = compute_rates(no_stdp)
print_rates(rates_no_stdp)
```

```
Firing rate nest                 : 11.01 ± 0.05 sp/s
Firing rate brian_exp_euler      : 12.10 ± 0.04 sp/s
Firing rate brian_exp_expeuler   : 11.73 ± 0.06 sp/s
Firing rate brian_delta_euler    : 12.02 ± 0.06 sp/s
Firing rate brian_delta_expeuler: 10.86 ± 0.04 sp/s
```

Model without connections

In [26]:
```
rates_no_conn = compute_rates(no_conn)
print_rates(rates_no_conn)
```

```
Firing rate nest                 :  9.92 ± 0.07 sp/s
Firing rate brian_exp_euler      : 10.90 ± 0.05 sp/s
Firing rate brian_exp_expeuler   :  9.89 ± 0.04 sp/s
Firing rate brian_delta_euler    : 10.93 ± 0.04 sp/s
Firing rate brian_delta_expeuler:  9.86 ± 0.05 sp/s
```

Plot results

In [27]:
```
def plot_activity(spks, bins, n_size, ax, lbl, between=True):
    cnts = [np.histogram(s, bins)[0] for s in spks]
    ca = np.vstack(cnts) / n_size*100
    mc = ca.mean(axis=0)
    sc = ca.std(axis=0)
    ax.plot(bins[1:], mc, label=f'{lbl}');
    if between:
        ax.fill_between(bins[1:], mc-sc, mc+sc, alpha=0.3);
    ax.set_xlim(0, 300);
```

In [28]:
```
def plot_set(data, bins, n_size, ax, cases, legend=True, between=True):
    for key, label in cases.items():
        plot_activity(data[key]['spikes_t'], bins, n_size, ax, label, between);
    if legend:
        ax.legend();
```

Spike activtity with delta synapses in NEST and Brian

In [29]:
```
cases_delta = {'nest': 'NEST',
               'brian_delta_euler': 'Brian (Euler)',
               'brian_delta_expeuler': 'Brian (Exp Euler)'}
n_size = 10000
bins = np.arange(0, 305, 1)

plt.figure(figsize=(10, 6), layout='compressed');
ax = plt.subplot(3, 1, 1)
plot_set(full, bins, n_size, ax, cases_delta)
ax.set_title('Full model')

ax = plt.subplot(3, 1, 2)
plot_set(no_stdp, bins, n_size, ax, cases_delta)
ax.set_title('Model without STDP')
```

```
ax = plt.subplot(3, 1, 3)
plot_set(no_conn, bins, n_size, ax, cases_delta)
ax.set_title('Model without recurrent connections');
```

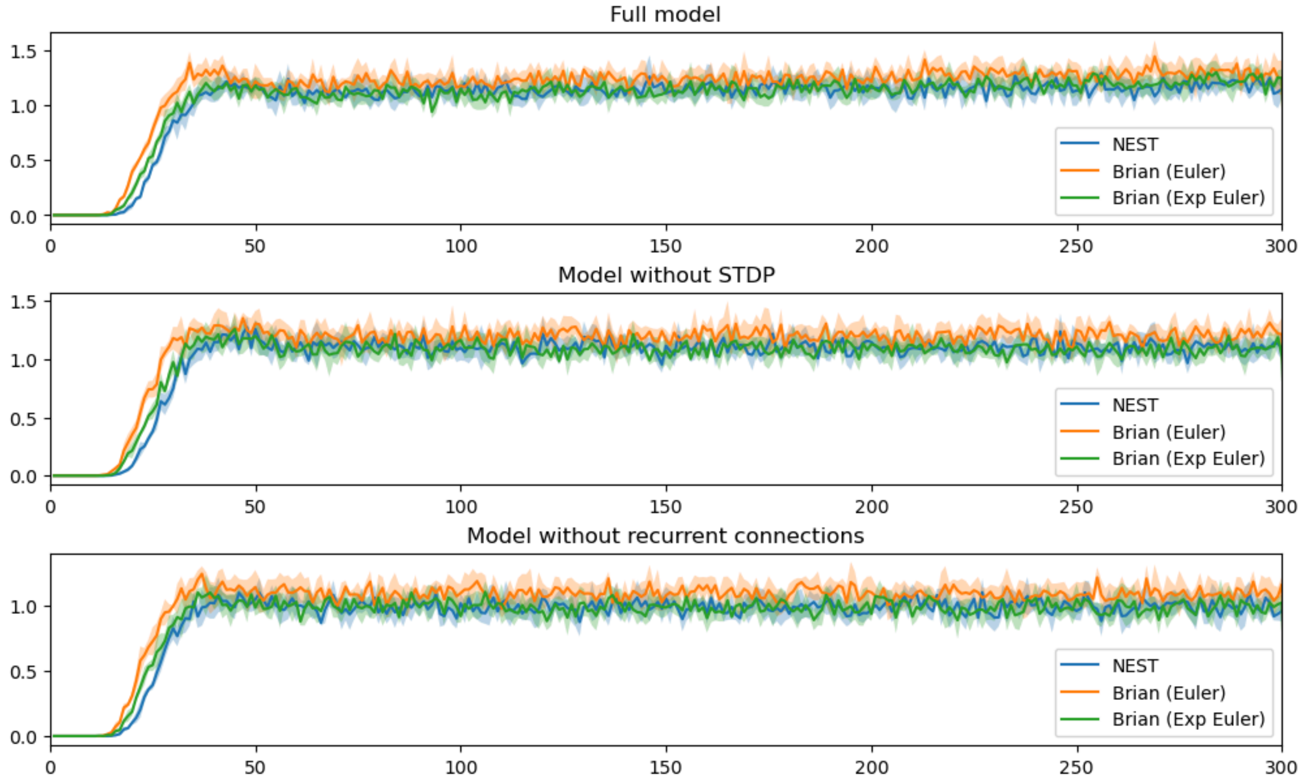

Spike activtity with delta synapses in NEST and exponential synapses in Brian

In [30]:

```
cases_exp = {'nest': 'NEST',
             'brian_exp_euler': 'Brian (Euler)',
             'brian_exp_expeuler': 'Brian (Exp Euler)'}
n_size = 10000
bins = np.arange(0, 305, 1)

plt.figure(figsize=(10, 6), layout='compressed');
ax = plt.subplot(3, 1, 1)
plot_set(full, bins, n_size, ax, cases_exp)
ax.set_title('Full model')

ax = plt.subplot(3, 1, 2)
plot_set(no_stdp, bins, n_size, ax, cases_exp)
ax.set_title('Model without STDP')

ax = plt.subplot(3, 1, 3)
plot_set(no_conn, bins, n_size, ax, cases_exp)
ax.set_title('Model without recurrent connections');
```

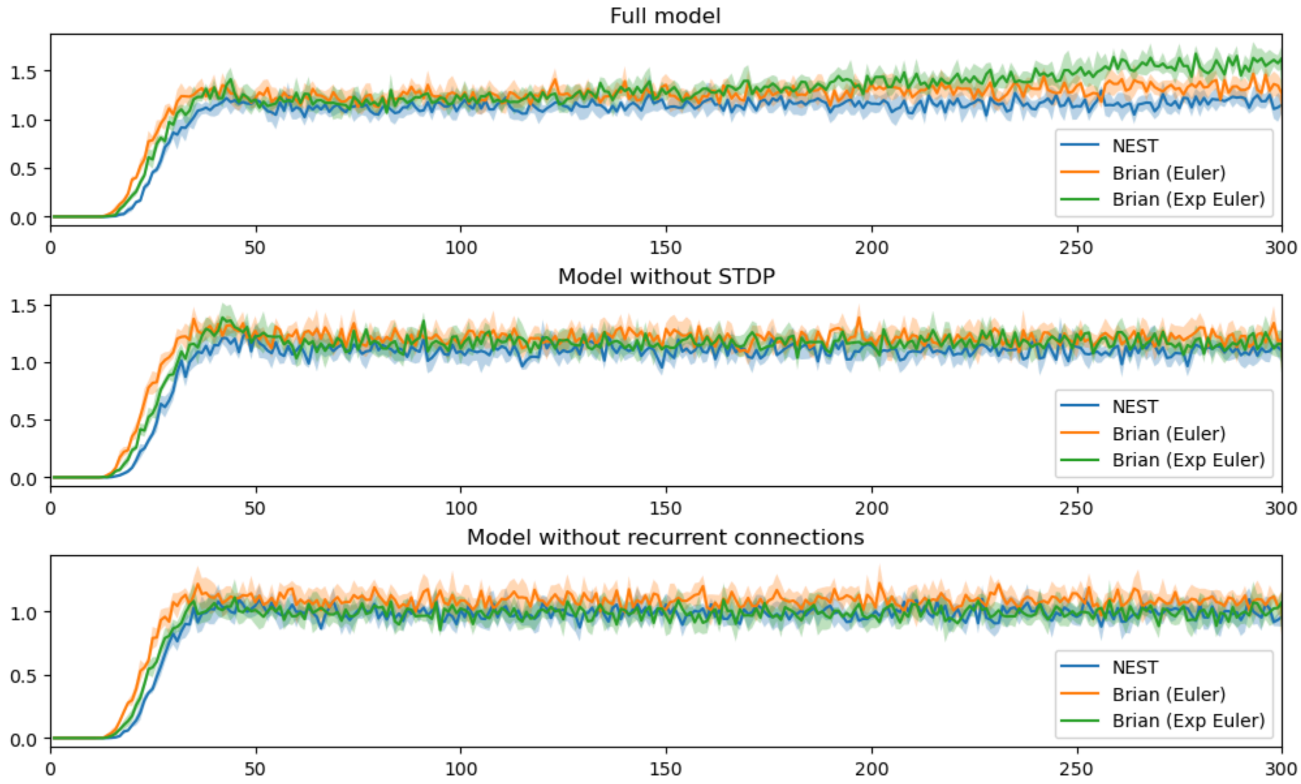

Figure for paper

Perform single simulation over 1000s to obtain membrane potential distribution (Python code from above)

In [31]:
```
_, V_exact, V_euler, sp_exact, sp_euler = sim(1000000)
```

In [32]:
```
hc_eu, be_eu = np.histogram(V_euler, bins=600, density=True);
hc_ex, be_ex = np.histogram(V_exact, bins=600, density=True);
```

Plot functions slightly modified from above for better control over line properties

In [33]:
```
def plot_pV(ax):
    ax.stairs(hc_ex, be_ex, label='Exact Sim', lw=2, ec=(1, 0.4, 0.), zorder=50);
    ax.stairs(hc_eu, be_eu, label='Euler Sim', lw=2, ec=(0.18, 0.55, 1.), zorder=0);
    ax.step(vv_exact, phi_exact*100, where='post', label='Exact Markov', lw=1, c='r', zorder=100);
    ax.step(vv_euler, phi_euler*100, where='post', label='Euler Markov', lw=1, c=(0., 0., 1.0),
            zorder=20);
```

In [34]:
```
def pplot_activity(spks, bins, n_size, ax, lbl, params):
    cnts = [np.histogram(s, bins)[0] for s in spks]
    ca = np.vstack(cnts) / n_size*100
    mc = ca.mean(axis=0)
    ax.stairs(mc, bins, **params, label=f'{lbl}');
    ax.set_xlim(0, 300);
```

In [35]:
```
def pplot_set(data, bins, n_size, ax, cases, legend=True):
    for key, (label, params) in cases.items():
        pplot_activity(data[key]['spikes_t'], bins, n_size, ax, label, params);
    if legend:
        ax.legend(fontsize='xx-small', loc='lower left', bbox_to_anchor=(0.1, 0.03), frameon=False);
```

In [36]:
```python
pcases = {
    'nest': ('NEST', {'lw': 2, 'ec': (1, 0.4, 0.), 'zorder': 100}),
    'brian_delta_euler': ('Brian/Eul.', {'lw': 2, 'ec': (0.18, 0.55, 1.), 'zorder': 10}),
    'brian_delta_expeuler': ('Brian/Exp Eul.', {'lw': 2, 'ec': (0.7, 0.3, 1), 'zorder': 0})}
```

In [37]:
```python
plt.rc('text', usetex=True);
fig, axd = plt.subplot_mosaic([['top'],
                               ['middle'],
                              ['bottom']],
                              height_ratios=[3, 1, 2], #[0.55, 0.15, 0.3],
                              figsize=(85/25.4, 150/25.4), layout="constrained")

ax = axd['top'];
plot_pV(ax);
ax.set_ylabel('$q(v)$');
ax.set_xticks(range(7));
ax.set_ylim(0, 1.15);
ax.legend(fontsize='xx-small', loc='lower left', bbox_to_anchor=(0.35, 0.75), frameon=False);

ix1 = ax.inset_axes([0.675, 0.55, 0.3, 0.35], xlim=[5.5, 6.1], ylim=[0, 0.2], xticks=[], yticks=[]);
plot_pV(ix1)
_, cl1 = ax.indicate_inset_zoom(ix1, edgecolor="black");
for cl in cl1:
    cl.set_visible(not cl.get_visible())
ax.text(-0.2, 1, 'A', transform=ax.transAxes, va='top', ha='right', fontsize='large', fontweight='bold')

ix2 = ax.inset_axes([0.9, 0.15, 1.8, 0.4], xlim=[0.9, 1.1], ylim=[0, 0.05], xticks=[], yticks=[],
                   transform=ax.transData);
plot_pV(ix2)
_, cl2 = ax.indicate_inset_zoom(ix2, edgecolor="black");
ix2.vlines([alpha_exact, alpha_euler], ymin=0, ymax=0.05, colors='gray', ls=':', zorder=-100);

ix3 = ax.inset_axes([0.5, 0.75, 1.5, 0.3], xlim=[-0.06, 0.06], ylim=[0.8, 1.12], xticks=[], yticks=[],
                   transform=ax.transData);
plot_pV(ix3)
_, cl3 = ax.indicate_inset_zoom(ix3, edgecolor="black");

ax4 = axd['middle']
ax4.plot(vv_exact, phi_euler/phi_exact-1, c='k');
ax4.set_xlabel('Membrane potential $v$ [mV]');
ax4.set_ylabel('$1-q_{{Eul}}/q_{{Exa}}$');
ax4.set_xticks(range(7));
ax4.text(-0.2, 1, 'B', transform=ax4.transAxes, va='top', ha='right', fontsize='large', fontweight='bold')

ax5 = axd['bottom']
bins = np.arange(0, 305, 1);
pplot_set(full, bins, 10000, ax5, pcases, legend=True);
ax5.set_xlabel('Time $t$ [ms]');
ax5.set_ylabel(r'Pop. activity [\%]');
ax5.set_yticks(np.arange(0, 1.51, 0.25));
ax5.set_xlim(0, 300);
ax5.text(-0.2, 1, 'C', transform=ax5.transAxes, va='top', ha='right', fontsize='large', fontweight='bold')

ix5= ax5.inset_axes([0.45, 0.05, 0.5, 0.5], xticks=[], yticks=[]);
pplot_set(full, bins, 10000, ix5, pcases, legend=False);
ix5.set_xlim(200, 250);
ix5.set_ylim(1, 1.5);
_, cl5 = ax5.indicate_inset_zoom(ix5, edgecolor="black");


plt.savefig('figures/markov_analysis.eps', bbox_inches='tight');
```

```
The PostScript backend does not support transparency; partially transparent artists will be rendered opaqu
e.
```

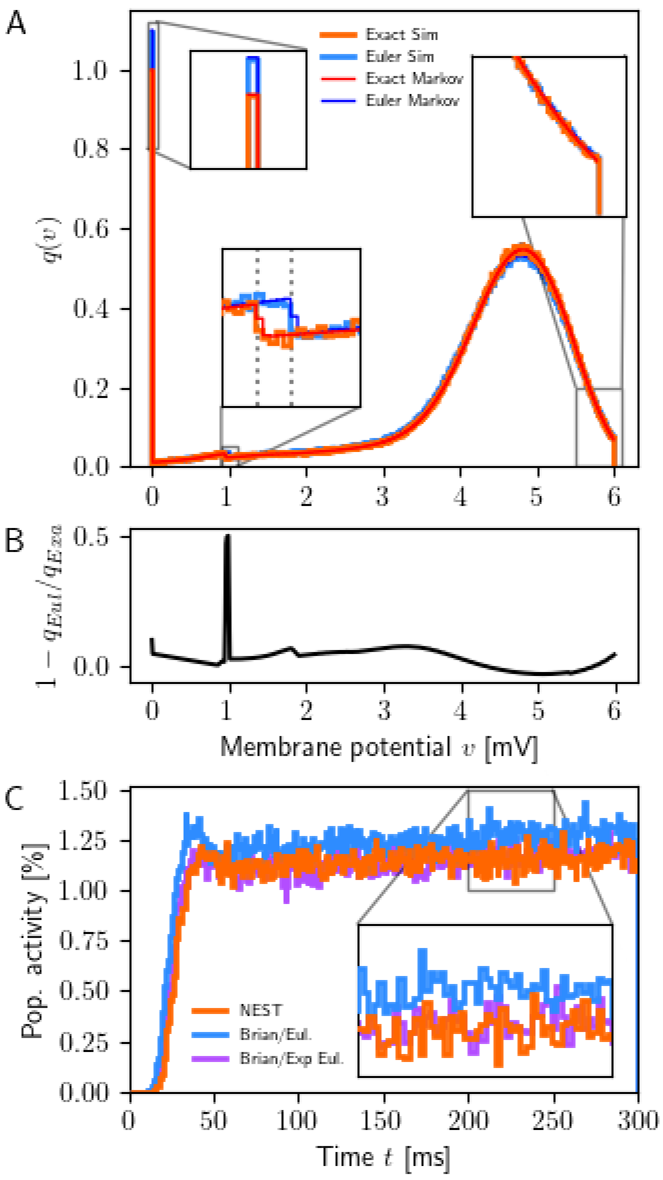

STDP analysis

Based on 1000 neuron network to avoid overly large weight matrices, single run only

In [38]:
```
if False:
    run_simset(1, 1000, 'rec_weights')  # smaller network, record weights
```

In [39]:
```
small = pickle.load(open('data/sim_1000_Conn_True_STDP_True.pkl', 'rb'))
```

Initial membrane potential distribution

In [40]:
```
cases_all = {'nest': 'NEST',
             'brian_delta_euler': 'Brian (Delta / Euler)',
             'brian_delta_expeuler': 'Brian (Delta / Exp Euler)',
             'brian_exp_euler': 'Brian (Exp / Euler)',
             'brian_exp_expeuler': 'Brian (Exp / Exp Euler)'}
plt.figure(figsize=(10, 4));
for name, data in small.items():
```

```
    data['w_ini'][0].hist(bins=1000, histtype='step', label=name);
plt.legend();
plt.xlabel('Synaptic weight $w$');
plt.ylabel('Counts per bin');
```

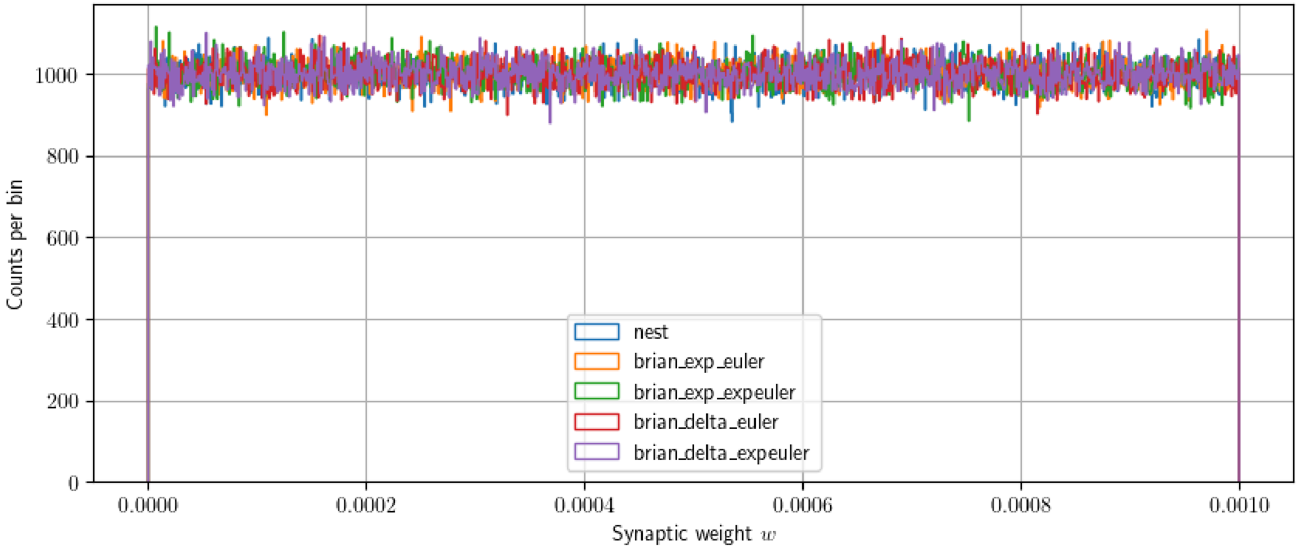

Final weight distribution

Expected weight distribution

- A synapse weight is increased by 0.001 if the pre- and post-synaptic neuron fire in subsequent time steps
- Since firing is rare (ISI approx 100 ms), we can treat pre- and post-synaptic spikes as independent
- The probability that a specific synapse is increased in a given time step is

$$p_{\text{STDP}} = \left(\frac{h}{r(\infty)}\right)^2$$

- In 300 ms simulation time there are $n_{\text{STDP}} = 299$ time steps with opportunity for plasticity
- Thus, the expected distribution of weights after a 300 ms simulation is given by the following binomial distribution

In [41]:
```
stdp_steps = 299
p_spike = rates_full['mean']['nest'] / h / 1000   # 1000 for ms->s
p_stdp = p_spike**2
expected_weight_dist = binom(stdp_steps, p_stdp)
```

In [42]:
```
wbins = np.arange(0, 0.0105, 0.001)
plt.figure(figsize=(10, 4));

plt.subplot(1, 2, 1)
for name, data in small.items():
    data['w_post'][0].hist(bins=wbins, histtype='step', label=name, log=True);
x_stdp = np.arange(0, 11.4, 1)
plt.gca().vlines(0.0005 + x_stdp*0.001, 0, expected_weight_dist.pmf(x_stdp)*1e6,
                 colors='k', linestyles='-', lw=3, label='Binomial prediction')
plt.ylim(1e-2, 1.5e6)
plt.legend();
plt.xlabel('Synaptic weight $w$');
plt.ylabel('Counts per bin');
plt.title('Synaptic weight distribution binned stepwise');

plt.subplot(1, 2, 2)
for name, data in small.items():
```

```
    data['w_post'][0].hist(bins=np.arange(0, 0.0105, 0.00001), histtype='step', label=name,
                           log=True, alpha=0.6);
plt.legend();
plt.ylim(5e-1, 1.5e4)
plt.xlabel('Synaptic weight $w$');
plt.ylabel('Counts per bin');
plt.title('Synaptic weight distribution in finer bins');
```

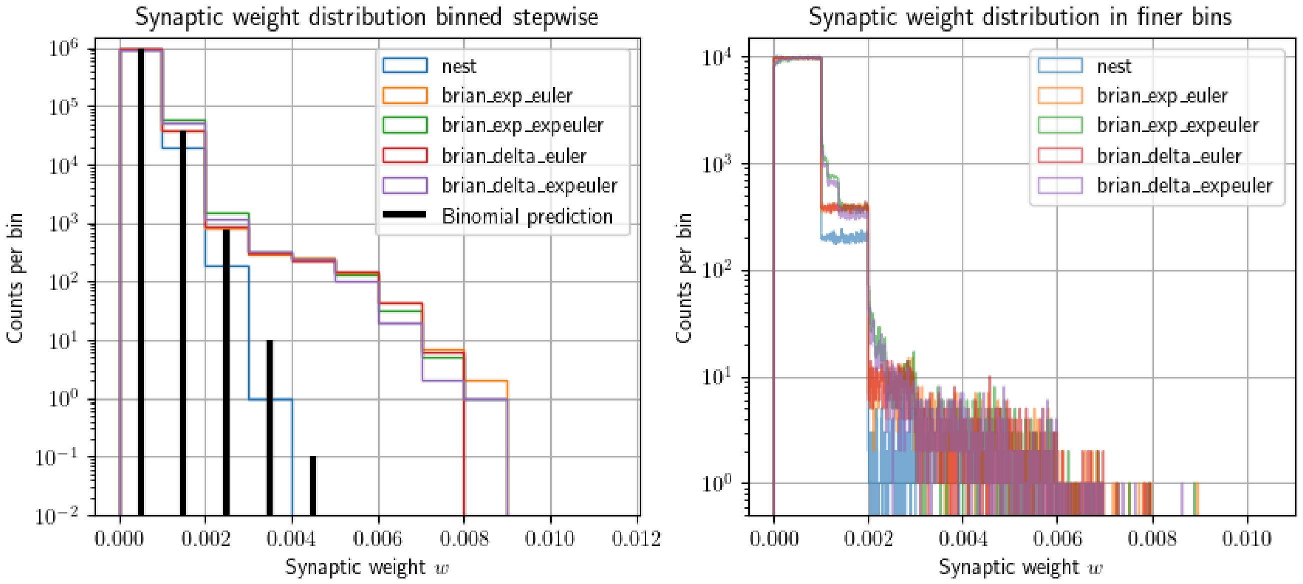

- Columns in the left graph represent from left to right synapses that have undergone no, one, two, etc weight increases.
- Initially, we only had a single flat column for weights in $[0, 0.001)$. Since weights always increase by 0.001 , all subsequent steps must also be flat.
- In the left graph, each column is 0.001 wide, thus by construction flat. In the right graph, we have 100 bins per step, more finely resolving the weight distribution.
- NEST (blue) shows too few neurons with weight increases. This is primarily due to the fact that a weight change in NEST first becomes measurable with the next spike of the postsynaptic neuron.
- Brian simulations show too many synapses with three and more weight increases. The reason for this is unclear.
- Brian using exponential Euler integration shows different weight distributions than with plain Euler (left graph) and resolving weights more finely, one sees that instead of flat stairs as expected we see transients in case of exponential Euler. This is most clearly visible in the $[0.001, 0.002)$ interval. The reason for this is that the `dspiked/dt = -spiked / (1*ms) : 1` equation in Brian only resets `spiked` instantaneously from one time step to the next when integrating with forward Euler.

Replay STDP development

- Given spike trains from actual simulations, we can replay the STDP process in Python by finding all situation in which a spike in one neuron is followed by a spike in another neuron in the next time step.
- To account for NEST's approach to effectuate the weight change caused by a pre-post spike pair only when the next spike passes through the synapse, we must only include a plasticity event if it is followed by a later presynaptic spike when replaying NEST simulations.

In [43]:
```
def stdp_replay(data, wbins, nest=False):
    """
    If nest is True, include pre-post pair only if followed by another pre spike.

    Returns histogram over weights and full matrix.
```

```
    """

    n_rep = len(data['spikes_t'])

    hists = np.zeros((n_rep, len(wbins)-1), dtype=int)
    W_all = []

    for rep in range(n_rep):
        s_t, s_id = data['spikes_t'][rep].values, data['spikes_id'][rep].values

        if nest:
            t_max = np.zeros(1000, dtype=int)
            for t, gid in zip(s_t, s_id):
                if t == 300:
                    continue
                t_max[gid-1] = t   # exploit that spikes for a given neuron are ordered

        W = np.zeros((1000, 1000))
        id_pre = []
        for t_post in range(1, 300):
            t_pre = t_post - 1
            id_post = s_id[np.argwhere(s_t == t_post)]
            if not nest or len(id_pre) == 0:
                use_id_pre = id_pre
            else:
                t_max_pre = t_max[id_pre-1]
                use_id_pre = id_pre[t_pre < t_max_pre]
            for gid_pre in use_id_pre:
                W[id_post-1, gid_pre-1] += 0.001
            id_pre = id_post

        hists[rep, :] = np.histogram(W.ravel(), bins=wbins)[0]

        W_all.append(W)

    return hists, W_all
```

Replay results

- We obtain the full replay matrix only for NEST to later explore it in detail
- For Brian, we focus on weight distributions and delta synapses
- We expect that the replay produces *exactly* the same weights and thus weight distributions as the simulation
- Note that because the simulations scripts provided by Vieth et al obtain random seeds from the system clock, detailed numbers below can vary if simulations are re-run.

In [44]:
```
wh_nest, W_replay = stdp_replay(small['nest'], wbins, nest=True)
wh_brian_euler, _ = stdp_replay(small['brian_delta_euler'], wbins)
wh_brian_expeuler, _ = stdp_replay(small['brian_delta_expeuler'], wbins)
```

In [45]:
```
n_stdp = (wbins[:-1]/0.001).astype(int)
n_binom = (1e6*expected_weight_dist.pmf(n_stdp)).astype(int)
pd.DataFrame.from_dict({'n_stdp': n_stdp, 'Theory': n_binom,
                        'NEST Sim': np.histogram(small['nest']['w_post'][0], wbins)[0],
                        'NEST Replay': wh_nest[0],
                        'Brian Euler Sim':
                            np.histogram(small['brian_delta_euler']['w_post'][0], wbins)[0],
                        'Brian Euler Replay': wh_brian_euler[0],
                        'Brian ExpEul Sim':
                            np.histogram(small['brian_delta_expeuler']['w_post'][0], wbins)[0],
                        'Brian ExpEul Replay': wh_brian_expeuler[0]
                       }).set_index('n_stdp')
```

Out[45]:

n_stdp	Theory	NEST Sim	NEST Replay	Brian Euler Sim	Brian Euler Replay	Brian ExpEul Sim	Brian ExpEul Replay
**0**	960847	979691	977684	960256	961308	946946	966858
**1**	38378	20121	22108	38156	38094	51173	32678
**2**	763	187	207	858	593	1199	462
**3**	10	1	1	302	5	320	2
**4**	0	0	0	225	0	236	0
**5**	0	0	0	152	0	103	0
**6**	0	0	0	45	0	20	0
**7**	0	0	0	6	0	2	0
**8**	0	0	0	0	0	1	0
**9**	0	0	0	0	0	0	0

- The NEST simulation and replay results show far more synapses with unchanged weight that expected (approx 978.000 vs 960.000). This is due to the fact that only pre-post spike pairings followed by another presynaptic spike with the simulation time are observable.
- In the NEST simulation, approximately 2000 more synapses (out of 1 million) remain unchanged compared to the replay. This turned out to be a bug in the code generated by NESTML for the synapse model (see https://github.com/nest/nestml/issues/1057, case 1).
- The Brian simulation results show long tails (four or more STDP changes), while the Brian replay results (based on the spikes simulated by Brian) show no more than three STDP changes. The latter is in accordance with the prediction from the binomial distribution. This may indicate an error in the Brian implementation of the STDP rule.
- Brian with exponential Euler shows results consistent with theory on replay, while simulation results show far too few synapses with unchanged weights (946.946 vs expected 960.832). This is due to the fact that equation

  ```
  dspiked/dt = -spiked / (1*ms) : 1
  ```
  in the Brian implementation of the plasticity rule when integrated with exponential Euler will not lead to an instantaneous, complete reset of `spiked`.

Analysis of NEST STDP discrepancy

- Why does NEST Sim show about 2000 more unchanged weights than NEST replay?
- We consider NEST replay here the ground truth.
- The weight matrix `W_replay` obtained from `stdp_replay()` contains all changes due to STDP, but not the intial values.
- We create a corresponding matrix from simulated data using the source and target indices stored with the weights (-1 to convert from 1-based neuron IDs in NEST to 0-based Python indices; rows: targets, columns: sources):

In [46]:
```
W_sim = np.zeros((1000, 1000))
W_sim[small['nest']['w_tgt'][0]-1, small['nest']['w_src'][0]-1] = ( small['nest']['w_post'][0]
                                                                     - small['nest']['w_ini'][0] )
```

In [47]:
```
deltaW = W_replay[0] - W_sim
too_many = np.argwhere(deltaW < -1e-6)
too_few = np.argwhere(deltaW > 1e-6)
print(f"Synapses with too many weight increases: {len(too_many):4d}")
print(f"Synapses with too  few weight increases: {len(too_few):4d}")
```

```
Synapses with too many weight increases:    0
Synapses with too  few weight increases: 2027
```

- In the simulations, we had no extra weight increase, but some 2000 increases are missing
- Thus, the NEST simulations missed some 2000 pre-post spike pairings
- There is no obvious pattern in the misses:

In [48]:
```
too_few[:50:10]
```

Out[48]:
```
array([[  0,  18],
       [  3, 202],
       [  7, 133],
       [ 10, 701],
       [ 21, 503]])
```

- We take the first case as an example, i.e., `[0, 18]`.
- Since these are target-source indicies, they correspond to target neuron 1, source neuron 19.
- We now use weight recorder data to look more closely at pertaining patterns

In [49]:
```
wr = small['nest']['wrec'][0].set_index(['targets', 'senders']).drop(['Unnamed: 0', 'receptors'],
                                                                      axis=1).sort_index()
```

In [50]:
```
tx, sx = too_few[0, :] + np.array([1, 1], dtype=int)
```

In [51]:
```
tx, sx
```

Out[51]:
```
(1, 19)
```

In [52]:
```
wr.loc[(tx, sx)]
```

Out[52]:

targets	senders	ports	times	weights
1	19	2250	52.0	0.000208
	19	2250	178.0	0.000208
	19	2250	229.0	0.000208

- Three spikes passed the 19 -> 1 synapse, the weight retained its initial value
- We now look at the spike times of the postsynaptic neuron, neuron 1

In [53]:
```
spks = pd.DataFrame.from_dict({'senders': small['nest']['spikes_id'][0],
        'times': small['nest']['spikes_t'][0]}).set_index('senders').sort_values('times')
```

In [54]:
```
spks.loc[tx].T
```

Out[54]:

senders	1	1	1	1	1
times	53.0	82.0	160.0	262.0	287.0

- After the sender (19) spiked at 52ms, the target (1) spiked at 53 ms, and this should have caused a weight increase by 0.001.
- In NEST, this weight increase should have been visible in the weight recorder entry for the spike from 19 to 1 at 178 ms.
- We note that the postsynaptic neuron spiked two more times (82 and 160 ms) before the next spike through the 19->1 synpse at 178 ms.

Good vs bad cases

- The following analysis depends on the 52-53 ms spike timing. For different simulation data, this may need to be adjusted. Choose the pair of spike times that should have led to stdp.
- We now look in the full spike data for all pairs of neurons in which one fired at 52 ms and the other at 53 ms and group them as follows:
  - *Good*: synapse weight has increased
  - *Bad*: synapse weight has not increased
  - *Latent*: synapse weight increase is not visible since no more spike through synapse after 53 ms

In [55]:
```
def group_events(spks, wr, pre_time):
    pre_spikes = spks.loc[spks.times==pre_time]
    post_spikes = spks.loc[spks.times==pre_time+1]
    pairs = [(post, pre) for pre in pre_spikes.index for post in post_spikes.index]

    res = {'good': [], 'bad': [], 'latent': []}
    for ix in pairs:
        elem = wr.loc[ix]  # all spikes between pre and post neuron
        tix = np.argwhere(elem.times.values == pre_time)  # index for spike at given time
        if tix < len(elem) - 1:
            if elem.weights.values[tix+1] > elem.weights.values[tix]:
                res['good'].append(ix)
            else:
                res['bad'].append(ix)
        else:
            res['latent'].append(ix)
    return res
```

In [56]:
```
gr = group_events(spks, wr, 52)
```

Good example 1

In [57]:
```
wr.loc[gr['good'][0]]
```

Out[57]:

		ports	times	weights
**targets**	**senders**			
**863**	**943**	117864	52.0	0.000998
	**943**	117864	224.0	0.001998

- The presynaptic neuron (943) fires at 52 and 224 ms
- The weight at 224 ms is increased by 0.001 compared to the value at 52 ms

In [58]:
```
spks.loc[863].T
```

Out[58]:

**senders**	**863**	**863**
**times**	53.0	268.0

- The postsynaptic neuron (863) fired at 53 ms
- Together with the spike at 52 ms from the presynaptic neuron, this explains the weight increase observed
- The postsynaptic neuron does not fire any new spike before the second spike of the presynaptic neuron at 224 ms

Good example 2

In [59]:
```
wr.loc[gr['good'][15]]
```

Out[59]:

targets	senders	ports	times	weights
606	19	2350	52.0	0.000398
	19	2350	178.0	0.001398
	19	2350	229.0	0.001398

In [60]:

```
spks.loc[606].T
```

Out[60]:

senders	606	606	606
times	53.0	185.0	266.0

- For the 19 -> 606 synapse, the spike pairing at 52/53 ms explains the weight increase observed at 178 ms
- The postsynaptic neuron does not fire between 53 ms and the next spike from the presynaptic neuron at 178 ms

Bad case 1

In [61]:

```
wr.loc[gr['bad'][0]]
```

Out[61]:

targets	senders	ports	times	weights
797	943	117799	52.0	0.000104
	943	117799	224.0	0.000104

In [62]:

```
spks.loc[797].T
```

Out[62]:

senders	797	797	797	797
times	53.0	93.0	182.0	281.0

- For the 943 -> 797 synapse, the postsynaptic neuron fires twice (at 93 and 182 ms) before the "readout" spike at 224 ms

Bad case 2

In [63]:

```
wr.loc[gr['bad'][15]]
```

Out[63]:

targets	senders	ports	times	weights
951	392	48948	52.0	0.000516
	392	48948	271.0	0.000516

In [64]:
```
spks.loc[951].T
```

Out[64]:

senders	951	951	951	951
times	53.0	176.0	201.0	286.0

- For the 392 -> 951 synapse, the postsynaptic neuron fires twice (at 176 and 201 ms) before the "readout" spike at 271 ms

Why NEST misses STDP events in some cases for the Vieth STDP rule

- Comparing the good vs bad cases above, it appears that the occurence of more than one post-synaptic spike between the pre-post pairing and the next pre-synaptic spike (aka readout spike) makes NEST miss an STDP event.
- This is due to the code generated by NESTML for the Vieth STDP rule.
- This has been logged [as a NESTML issue](See https://github.com/nest/nestml/issues/1057) and is now corrected in NESTML.

In [ ]:
```

```
